# Energy Solutions for Wearable Sensors: A Review

**DOI:** 10.3390/s21113806

**Published:** 2021-05-31

**Authors:** Guoguang Rong, Yuqiao Zheng, Mohamad Sawan

**Affiliations:** 1 CenBRAIN Lab., School of Engineering, Westlake University, Hangzhou 310024, China; rongguoguang@westlake.edu.cn (G.R.); zhengyuqiao@westlake.edu.cn (Y.Z.); 2 CenBRAIN Lab., Institute for Advanced Study, Westlake Institute for Advanced Study, Hangzhou 310024, China

**Keywords:** energy sources, power solutions, wearable devices, biosensors, health monitors, scavengers, harvesters

## Abstract

Wearable sensors have gained popularity over the years since they offer constant and real-time physiological information about the human body. Wearable sensors have been applied in a variety of ways in clinical settings to monitor health conditions. These technologies require energy sources to carry out their projected functionalities. In this paper, we review the main energy sources used to power wearable sensors. These energy sources include batteries, solar cells, biofuel cells, supercapacitors, thermoelectric generators, piezoelectric and triboelectric generators, and radio frequency (RF) energy harvesters. Additionally, we discuss wireless power transfer and some hybrids of the above technologies. The advantages and drawbacks of each technology are considered along with the system components and attributes that make these devices function effectively. The objective of this review is to inform researchers about the latest developments in this field and present future research opportunities.

## 1. Introduction

Over the years, wearable sensors have attracted considerable attention due to their ability to offer constant and real-time physiological information. Wearable sensors dynamically and noninvasively measure biochemical markers found in biological fluids, including sweat, tears and interstitial fluids. Recently, the non-invasive monitoring of biomarkers through the use of biosensors for both healthcare and sports analytics has become a research hotspot [1]. Researchers have miniaturized composite biosensors, transmission systems and microfluidic sampling platforms so that these techniques can be integrated with flexible materials to enhance wearability and facilitate corresponding operations [2].

**Figure 1:**
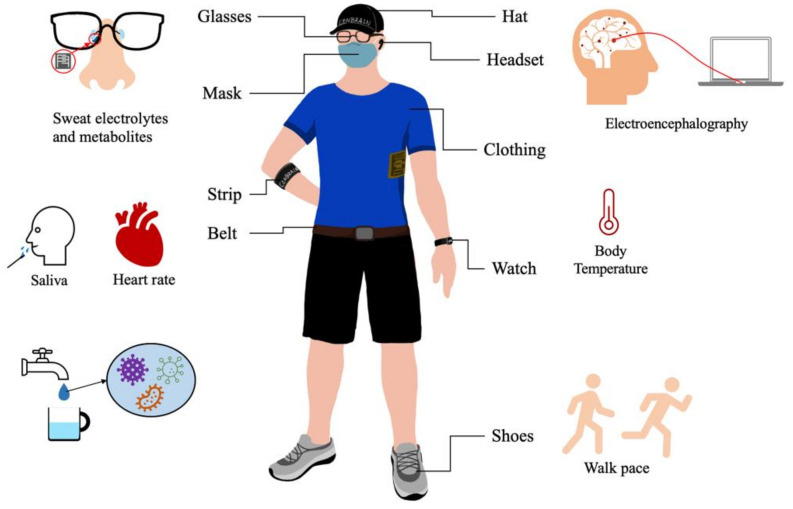
Wearable medical and healthcare devices for various regions of the body.

Wearable sensors have various clinical uses and are capable of sensing changes in human physiology, biochemistry and motion, which is required for both diagnostics and treatments in sports applications (Figure 1). Additionally, these technologies can play a vital role in treating chronic illnesses by realizing continuous drug monitoring, which is a promising technique intended to replace current therapeutic drug monitoring strategies [3]. Physicians have used wearable sensors in several formats that are built to be attached to the human body to measure bio-signals. Biosensors can be embedded in glasses [4], contact lenses [5], mouth guards [6], clothing [7], wrist bands [8] and many other products. Wearable sensors can measure cardiovascular activity [9], body signals such as heart rate [10], blood pressure and respiration rate [11], and sweat content and interstitial fluid [12], which includes glucose, sodium and potassium levels. There have also been studies of the development of wearable electroencephalography (EEG) devices and using deep neural networks for EEG data analysis [13]. Wearable technologies based on multimodal EEG-functional near-infrared spectroscopy (EEG-fNIRS) have been used to monitor physiological parameters related to strokes, which can help prevent stroke-related disability or death [14].

Wearable sensors have strong potential for improving current medical systems, as they are able to provide extensive monitoring of various physiological functions and highly efficient economical services in a variety of fields. However, wearable sensors need appropriate energy sources, given that their maintenance depends on a continuous, stable and sufficient supply of power. Different forms and applications of wearable sensors require different amounts of energy to work. Therefore, it is of great importance to design and select reliable energy supply systems with suitable structures, sizes and energy densities. Wearable sensors generally use batteries as a power source. Battery technology is very mature, and various power management strategies for prolonging battery life have been proposed [15]. However, the use of batteries to power wearable sensors is associated with risks such as leakage of toxic substances and limited device lifetimes. An alternative solution involves wearable sensors harvesting energy from the human body or from the ambient environment. There are a number of technologies that can be used to harvest energy, including photovoltaic (PV) or solar cells, thermoelectric generators (TEGs), piezoelectric nanogenerators (PENGs), triboelectric nanogenerators (TENGs), biofuel cells (BFCs), electromagnetic generators (EMGs) and radio frequency (RF) harvesters. These technologies can be used in either single or hybrid formats [16]. When applied to wearable sensors, all of these approaches need to be well designed in terms of their structure, size, power density and energy output according to the application of interest. At the same time, these power generators need to conform well to the human body and be flexible, durable, stable, and non-toxic.

The remainder of this paper is composed of four sections. Section 2 discusses the methods most widely used to power biosensors, including batteries, solar cells, TEGs, BFCs and nanogenerators. Section 3 introduces three hybrid-generator systems and discusses their properties and performance. Section 4 compares the different types of generator systems and their performance parameters, and Section 5 draws conclusions with regard to the main research fields for which power generators are of interest and offers future directions of study. Table 1 summarizes the parameters used in this article.

## 2. Power Supply Solutions for Wearable Sensors

**Figure 2:**
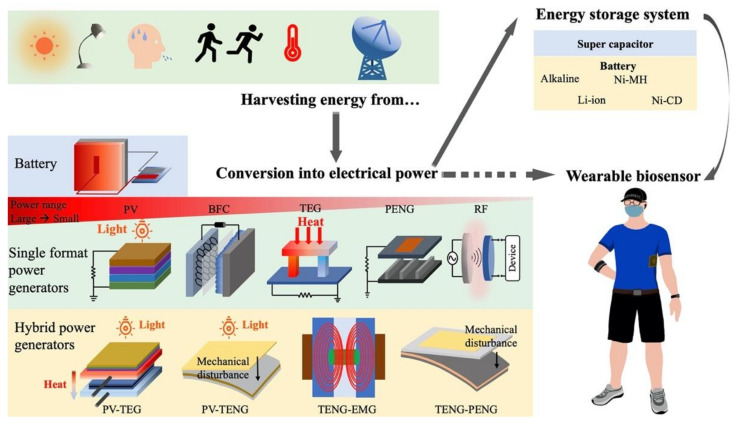
Schematic flow chart of self-powering smart wearable sensors. Harvesting energy from various energy sources, converting into electric power, storing energy in storage section and powering wearable sensors. PV: photovoltaic; BFCs: biofuel cells; TEGs: thermoelectric generators; PENGs: piezoelectric nanogenerators; TENGs: triboelectric nanogenerators; RF: radio frequency; EMGs: electromagnetic generators.

Available biosensors and wearable sensors under development require various power levels to be delivered from different sources of energy. Figure 2 shows some of the sources of energy that can be harvested, converted, and used to supply power to wearable sensors. Energy collected from light, heat, radio waves, or vibrations can be converted into electrical energy to power wearable sensors. The main energy solutions for wearable sensors are reviewed in the following sections.

### 2.1. Batteries

Wearable technologies, similar to any embedded system or portable device, require batteries to operate, and consumers would like smaller and thinner gadgets with batteries that can last for an extended period of time. Over the years, a number of different kinds of batteries have been introduced with the intention of meeting the power requirements of various wearable sensors.

Alkaline batteries, available since the 1960s, have been tested and widely used. These batteries are safe and easily replaceable. The main types of alkaline batteries are referred to as AA and AAA. These batteries are also available in coin cells and have a 1.5 V standard voltage and an 11.6 mm diameter and 5.4 mm height [17]. However, alkaline battery voltages drop with continued use; a steady and stable voltage is not achievable, and the voltage drops sharply near the end of the battery’s life. Nickel-metal hybrids are an alternative class of batteries that can be used to power wearable sensors. These types of batteries are rechargeable and have a capacity that is three times that of nickel-sodium batteries, with a value approaching that of lithium-ion batteries [15]. Lithium-ion and lithium-ion polymer batteries are the most commonly used batteries for wearable sensors. These batteries use Li-polymer cells or Li coin cells to make the batteries rechargeable and sustain power for an extended period of time to secure the normal operation of the wearable sensor [18].

Table 2 summaries typical batteries that can be used for wearable sensors. From this table we understand that lithium-ion batteries have a high working voltage, high specific energy, high specific power, long life cycle and very low self-discharge rate (2% per month) compared to other kinds of batteries. Table 3 compares some low-modulus and flexible substrate wearable sensors powered by different batteries. These sensors can be used to detect molecular or physical parameters such as pressure. In general, batteries can simplify device structure and size, power biosensors directly without the need for complex circuits [19].

Regardless of the type of battery used to supply energy to wearable sensors, whether it is a lithium-ion or alkaline battery, various designs have been implemented to ensure batteries fit user needs with regard to keeping wearable sensors safely powered [26]. However, the high toxicity of battery electrolytes is a disadvantage that cannot be ignored. On the other hand, wearable sensors have large limitations in size. Thus, in order to minimize device size, large and rigid battery packs are not expected. For some applications, batteries eventually may need to be replaced if they are of low capacity, and will run out of energy sooner or later in real applications. Devices powered by batteries require frequent charging, which can bring inconvenience. Therefore, except for enhancing the battery capacity, energy solutions that can provide an alternative power supply are being developed, such as energy harvesting technologies. On the other hand, although many studies are searching for alternatives to batteries, batteries and supercapacitors can also be combined in an energy harvesting system as the energy storage section. Therefore, optimizing battery performance inside wearable sensors is still a topic worth studying.

### 2.2. Solar Cells

Substantial attempts have been made by researchers to replace batteries as an energy source for numerous applications. Solar cells have been introduced to replace batteries to make devices lighter and more efficient [27]. Solar energy has various benefits in maintaining wearable device functionality. Solar cells can charge wearable devices through a USB connection integrated into a piece of cloth, which is very convenient in constructing different modules of devices via a standard interface, making devices more user-friendly and reducing their power demand. Since the introduction of miniaturized solar cells, it has become possible to generate power by solar energy in a variety of wearable objects. Thus, the use of wearable sensors has become more convenient than ever before [28].

Over the years, solar cells with reduced lengths and diameters have been synthesized; for example, the most recently developed solar cells are 3 mm in length and 1.5 mm in diameter, making them lighter and more suitable as a power source for wearable devices [29]. Solar-powered wearable devices are similar to any other textile but contain fibers with miniaturized cells that create electricity. This kind of flexible solar cells have been proved to be of high flexibility and durability. Table 4 provides some typical examples of these flexible textile solar cells. It has been found that 200 miniature solar cells embedded in a 20 cm^2^ section of fabric can yield a power of up to 43.5 milliwatts, which is sufficient to power a mobile phone [30].

Solar energy can also help optimize the performance of energy storage modules and thus can be more stable and adaptable for wearable electronics. Researchers used graphene oxide as solar-thermal conversion section to improve the low-temperature resilience of a supercapacitor [38]. In addition to high flexibility and scalability, this new design of a fiber-based supercapacitor can absorb and make use of sunlight energy to guarantee operation under extreme low temperatures (0 ℃), and thus is a promising choice of a combined energy harvesting and storage device for wearable and portable biosensors.

Perovskite solar cells are a class of solar cells that offer an efficient, flexible and lightweight energy solution for wearable electronic sensors. These solar cells are thin, flexible and very popular in, for example, portable electric chargers and wearable electronic textiles due to their convenience and versatile functionalities [39]. Flexible perovskite solar cells with a novel light absorber have been developed. An efficiency of 10.2% has been achieved by introducing a 3 µm-thick layer of this absorber. In other studies, research groups have succeeded in fabricating perovskite solar cells at a low temperature (130 °C) using another kind of light absorber [40]. These solar cells exhibit a higher power conversion efficiency (PCE) (15%) than those of optimized single-junction flexible organic solar cells. The most recent applications of perovskite solar cells based on organic light-absorbing halides have been shown to be economically and practically viable [41]. These solar cells are fabricated at low temperatures and can achieve PCEs above 16%. Researchers also studied bendable solar cells that can be applied to highly diffuse light environments. Jaehyun Park and his group build an analytical model in quantifying the relationship between harvested energy and the radius of cell curvature. The energy collected is increased by up to 25.0% under their proposed model [42]. Another work aimed at applying bending silicon photovoltaic cells for lighting poles. The authors studied the influence of illumination type (direct or diffuse irradiance), geometry condition (flat, half-bent or full-bent) and dye concentration on cell energy-harvesting performance [43]. The results show that the bent photovoltaic cells are less likely to be influenced by incoming irradiance angle compared with flat ones, and they also show stable performance under bad weather conditions. However, surface loss for the bending group was larger than that of the flat group. Although the authors only mentioned the application of their bent solar cell in architecture, it is still very promising for wearable sensors.

Table 4 summarizes the parameters of some high-PCE solar cells with high durability and stability. High flexibility can be significant in reducing wearing inconvenience, thus can benefit the commercialization of wearable sensors and their power supplying systems. To realize required flexibility, cell substrate and electrodes should be carefully designed. These solar cells can realize competitive PCE and output voltage levels for many portable electronic devices. They are suitable for wearable sensors without complicating the whole system. Note that all the experiments listed in Table 4 are done under illumination conditions of AM 1.5 G (100 mW $\cdot {\mathrm{cm}}^{-2}$).

Besides flexibility and stretchability, another important factor that can greatly affect the performance of solar cells is light intensity. Solar cells need further optimization to enhance their indoor performance, as the weather is not controllable and users spend most of their time inside buildings. It is worth mentioning that presently, many studies have been devoted to improving the indoor performance of solar cells [44,45], known as indoor photovoltaics. One work studying a photovoltaic-thermoelectric hybrid energy generator aims at achieving satisfactory self-powering ability in indoor environments when used in wearable devices [46]. Lee et al. tested the indoor performance of a perovskite photovoltaic cell under 200–1000 lux of illumination conditions. This cell realizes 18 $\mu W\cdot {\mathrm{cm}}^{-2}$ power density under 200 lux cool white light, and the fill factor kept stable among the light intensity range [47]. Biswas et al. enhanced the PCE of indoor organic solar cell by studying the doping concentration of the cell’s hole extraction layer. By testing under a 500 lx LED light, this cell realizes 8.1% PCE level [48]. Park and his group also proposed an indoor organic solar cell with very high PCE of up to 42% under indoor light conditions. After 1500 cycles of bending tests, this organic solar cell can maintain 84% of initial PCE [49]. All these works are aimed at improving the electrical performance of solar cells under low light environments and have made great progress.

Although solar cells exhibit higher PCEs than other types of energy generators [16], their efficiencies can be greatly reduced with increasing temperature [50]. The temperature coefficient of PV cells depends greatly on the type of material used [51]. Additionally, because some textile-based solar cells may suffer severe reductions in PCE following fatigue [52], the mechanical properties of solar cells should also be seriously taken into account. For example, when applied to wearable devices, these flexible devices face more complex body motions compared with experimental conditions. How to avoid material failure and performance degradation has become a problem that cannot be ignored. On the other hand, the temperature of solar cells can exceed the temperature of the human body significantly. For example, one experiment conducted by a research group tested the performance of organic solar cells under temperatures up to 85 °C [53]; thus, cooling systems may be needed, and methods that utilize wasted heat should be considered.

### 2.3. Thermoelectric Generators

TEGs are another type of energy source that can be used to power wearable devices. TEGs last for a long time, produce no noise, and generate the energy needed to directly power wearable devices by converting heat into electrical energy. TEGs have been shown to be effective in powering wearable sensors by using waste heat from the human body. The human body is a great portable source of energy, generating up to 58.2 W/m^2^ in waste heat at rest [54]. Even a small percentage of this waste heat is sufficient to power most low-energy wearable devices without the need for batteries as a backup energy source. Advancements in TEGs have allowed for more efficient power conversion. Thinner devices can reduce the profile and complexity of devices and remove the need for regular changes or swaps of energy cells. Additionally, TEGs can be directly integrated with wearable textiles [7]. Wearable sensors that operate with body heat can be used for long-term monitoring of human vitals and chronic illnesses. Human body heat-powered sensors have been used to monitor glucose levels and have been applied in hearing aids and accelerometer-based rehabilitation devices [55].

Thermoelectric energy has some advantages over other wearable sensor power sources. For example, the conversion of mechanical energy into electrical energy requires the user to be active, which may not be possible for elderly or bed-ridden individuals. Additionally, when users are exposed to low-lighting conditions, solar cells may not function properly. On the other hand, TEGs yield constant power as long as a difference between the temperature of the skin and the ambient temperature exists, which is typical for most practical conditions [56].

TEGs utilize the Seebeck effect to convert heat into electrical energy. When dissimilar materials, for instance, metals or semiconductors, which are n-type and p-type components, have junctions at different temperatures, the carriers of electrons and holes will move to the cold ends [57]. The Seebeck effect results in the creation of electric fields in both materials that are proportional to the temperature gradient. If there is a circuit connection, the current is able to flow [58]. TEGs are constructed by introducing a heat sink between the n- and p-type semiconductors and the heat source.

People generate heat as a result of metabolic functions, which serve to maintain core body temperature. The body temperature required for humans to maintain standard functionalities is approximately 37 °C. Heat exiting the skin is transferred to the ambient environment through convection and radiation at 1~10 mW cm^−2^. The rate at which heat is transferred depends on which body part is being considered. For example, muscles act as insulators, while arteries exhibit the highest heat-transfer efficiency of any body part. Clothing can obstruct heat transfer, leading to an average body heat transfer rate of approximately 5 mW cm^−2^ [59]. For accurate heat measurements, a body part such as the radial artery in the wrist may be targeted since it has a heat flow amounting to approximately 25 mW cm^−2^ at room temperature [59].

Electric generators that use body heat as an energy source may experience some shortcomings as a result of an insufficient conversion of body heat to electricity. For efficient conversion, there is a need for thin TEGs that do not consume much energy. Additionally, in some applications, there is a need for flexible wristbands with TEG modules that capture sufficient accelerometer data from a user [59].

Furthermore, industry has introduced flexible and organic TEGs with roll-by-roll methods. This is an efficient manufacturing process utilizing the rolling of different layers, which has led to the increased availability and reduced cost of TEGs. Better design in structure can improve the flexibility and durability of TEGs. Researchers have proposed highly flexible thermoelectric (TE) devices that can be integrated on arm bands. The output power density can reach $5.60\;\mu W\cdot {\mathrm{cm}}^{-2}$ by harvesting human body heat energy [60]. Table 5 lists some examples of flexible TEGs that function as a result of temperature differences between the human body and the environment. Usually, the temperature difference between the hot and cold side for wearable sensor should not be too large for general applications. Sun et al. prepared a fiber-based TEG that can be directly woven into textiles [61]. Additionally, a TEG-powered bracelet that can simultaneously monitor the temperature, humidity and motion of the human body has been reported [62]. Furthermore, the voltage increases linearly with increasing temperature difference ΔT, and the output power is positively correlated with ΔT [63].

### 2.4. Radio Frequency Energy Harvesters and Wireless Power Transfer (WPT)

RF harvesters, which use wireless power, offer an energy solution for wireless sensor networks [67]. RF technology harvests energy from the surrounding or dedicated energy sources. RF harvesters are attractive for many applications. Unlike other energy harvesters, such as solar cells or chemical generators, RF harvesters offer a continuous and controllable source of power, which makes them desirable for applications that require higher levels of energy [68]. Additionally, RF signals have been used to carry wireless information in wireless communications and can be utilized for sensor data transmission.

Depending on the WPT range, RF power transfer can be categorized as near-field inductive and capacitive coupling power transfer, ultrasonic power transfer, and mid- or far-field electromagnetic power transfer. Near-field inductive coupling is relatively mature and has been used to power cochlear implants [69]; however, careful alignments are needed to generate high power. Near-field capacitive coupling is used to power flexible patches, but the associated power transfer efficiency (PTE) drops dramatically when the transmitting coil and receiving coil are separated. Ultrasonic energy transfer is limited by large PTE fluctuations. Mid-field and far-field RF harvesters are less efficient when the frequency exceeds tens of gigahertz (GHz). Table 6 summarizes RF harvesters used to generate power for wearable sensors. Their PTE ranges from 5% to 90%, dependent on input power.

Over the past few years, WPT and data transfer have been investigated. Since these transfers are both enabled by RF signaling, many scholars have investigated simultaneous wireless data transfer and power transfer, which combines the two techniques [70].

There are also some problems in applying RF harvesters to power wearable sensors. For example, RF energy harvesting cannot generate enough power without perfect alignment in nearfield systems. Far-field RF harvester systems have low PTE inherently and may not satisfy the power requirements of wearable devices. One of the main concerns of RF harvesters is their capability to provide enough energy. On the other hand, RF harvesters need wake-up power, given that Complementary Metal-Oxide-Semiconductor (CMOS) transistor has a threshold. Although RF energy seems to act as a desirable and reliable source of energy for wearable devices, there is still room for constant advancement.

### 2.5. Biofuel Cells

BFCs are another energy recovery method used for wearable sensors. A fuel cell refers to an electrochemical cell wherein current is generated by reactions occurring between the chemical species flowing into the cell at the anodic site and the oxidant at the cathodic site [77]. Fuel cells are different from standard batteries, as they can produce continuous energy as long as the reactants are present. While there are various fuel cell forms, the most commonly used fuel cell involves a proton-exchange membrane [78]. In this type of fuel cell, a membrane separates the fuel and oxidant, allowing only protons produced at the anodic site to cross the membrane and minimizing the amount of oxidant present at the cathodic site. Electrons generated at the anode cannot pass through the membrane to reach the cathode; as such, they have to follow an alternative path, which results in the generation of current. The use of fuel cells to power wearable sensors has a number of advantages. The most important advantage is that the presence of reactants inside the fuel cell makes it unnecessary to replace the batteries [79]. In addition, elderly or bed-ridden individuals may utilize wearable sensors powered by BFCs using reactants available in human body, such as glucose or lactic acid.

When BFCs are used to power wearable sensors, the power supply can be combined with biosensing to simplify the design. Epidermal BFCs have been used to oxidize lactic acid in sweat to generate energy [80]. The energy generated from sweat helps the biofuels create ten times more energy per unit area than any other biofuel used in wearable sensors [81]. Jia et al. [82] prepared a BFC with bridge and island structures that was flexible, stretchable, and compatible with wearable sensors. This BFC contained two dotted rows linked by spring-shaped structures. Half of the dots comprised the anode, and the other half comprised the cathode. In addition, the spring-like structures between the dots stretched and bent without breaking or changing the initial structure of either the cathode or anode. The island and bridge structures were prepared from gold using lithography [83]. Then, researchers used a screen-printing strategy to place biofuel layers on top of the anode and cathode dots. Even though the approach was proven effective in producing energy, it was difficult to identify the amount of energy that these fuel cells could create per unit area. Thus, there is a need to quantify the amount of material to use and the combination ratio of different materials since these factors determine the amount of power generated [84]. Researchers are also trying to enhance the flexibility of biofuel cells. Shitanda et al. proposed a lactate paper-based biofuel cell [85] that can be very promising in wearable applications. It can provide ~3.4 V open circuit voltage (OCV) with six cells in series and 4.3 mW output power with a 6×6 cell array. Yin and his group demonstrated a flexible bracelet BFC that can collect sweat and utilize lactate to power wearable devices. It can generate 74 μW maximum output power and 0.39 V OCV at 20 mM lactate solution [86].

The biofuel cell array is proved to be of high performance in powering devices with low power cost. Table 7 lists some examples of BFCs that are or can be used to power wearable sensors. In general, BFCs are highly biocompatible, with lower fabrication costs. They make use of biochemical substances to supply power for wearable sensors. Recently, more BFCs with a high flexibility and small size have been investigated. However, BFCs require further studies to enhance their PCE. On the other hand, their output power relies heavily on analyte concentration.

### 2.6. Kinetic Energy Harvesters

Energy harvesters (EHs) are another type of energy source used to power wearable sensors. EHs function by trapping and accumulating vibrational energy produced by either human body movements or natural phenomena. ESs are considered green because they are biocompatible and environmentally friendly [92]. ESs provide low voltages and are suitable for applications requiring low power.

EH technology uses various techniques. The most important technique involves kinetic energy harvesting, where human motion is converted into energy. Kinetic energy harvesters can make use of vibration or motion to generate electrical power. They are classified by their transduction mechanisms: electromagnetic, electrostatic, piezoelectric and triboelectric [93].

#### 2.6.1. Electromagnetic Kinetic Energy Harvesters

An electromagnetic kinetic energy harvester contains electromagnetic transducers that can generate an electromotive force in response to changes in the external magnetic flux of a closed-loop circuit, by which electrical power is generated. Influx fluctuations can also be induced by, for example, making a circuit rotate around an axis, which changes the surface aligned with the magnetic flux [94]. Seiko has used this approach in its quartz wristwatch, which can self-charge through wrist motion due to energy transfer from an oscillating weight to a magnetic rotor attached to the watch’s coil. Kinetic harvesters can also have a charge pump circuit with various multiplicative factors to increase the battery voltage [95]. Wang et al. proposed an electromagnetic kinetic energy harvester that can convert walking, running and jumping mechanical energy to electrical power [96]. It is sensitive to vibrations with a frequency less than 100 Hz and is very small in size (10.58 × 2.06 × 2.55 ${\mathrm{mm}}^{3}$). This generator can provide 98.3 $\mu W\cdot {\mathrm{cm}}^{-3}\cdot g^{-1}$ normalized power density and 43.7 μW average output power.

#### 2.6.2. Electrostatic Kinetic Energy Harvesters

Electrostatic energy harvesters generate electrical power based on electrostatic induction. They contain a variable capacitor composed of two electrodes. External vibration disturbance can change the capacitor’s overlapping area, which leads to capacitance change. The devices resonate in response to this vibration disturbance and generate electricity [97]. The output power level is proportional to the harvesters’ operating frequency. Therefore, to gain maximum electrical power, the harvesters are expected to work at resonance.

An electrostatic kinetic energy harvester is able to greatly reduce the size of kinetic energy harvesters, which is very competitive in wearable devices. Microelectromechanical System (MEMS) electrostatic energy harvesters produce capacitance variation by mechanical vibrations and are typically designed in comb format [98]. MEMS electrostatic energy harvesters consist of a central mass and attached parallel electrostatic transducers. Hassana et al. designed a MEMS kinetic energy harvester generating an average output power of up to 195 nW at only 6 × 7 ${\mathrm{mm}}^{2}$ in size [99]. Lu’s group demonstrated a model that can predict the frequency performance of a MEMS kinetic energy harvester and concluded that bias voltage and stopper position can be designed to control the harvester power performance [100]. In addition to theoretical models, the author proposed a paper-based electrostatic kinetic energy harvester with a thickness less than 1 mm. It can provide 45.6 μW maximum output power with 16 MΩ load resistance [101]. In general, with further study and experiments, electrostatic kinetic energy harvesters with soft substrate are very promising in wearable sensors due to their small device size.

#### 2.6.3. Piezoelectric Nanogenerators

PENGs harvest energy by converting kinetic energy into electrical energy through the actions of nanostructured piezoelectric materials. A substantial amount of mechanical energy can be harvested from a variety of biological functions, including fluid flow, walking, heartbeats, breathing and muscle movements [102]. As such, PENGs, which convert mechanical stress into electrical charges, are the most promising energy harvesters for microsystems.

PENGs can be attached under shoes to generate power from leg motion [103]. In addition, a highly sensitive PENG can generate an output voltage near 11 mV from the vibration of a human throat when speaking [103]. PENGs have also been used to generate power for artificial skin, which can monitor the health condition of individuals [104].

The electric energy supplied by PENGs can be used to energize systems with power consumption requirements ranging from microwatts to milliwatts, which is a suitable range for wearable sensors. Experiments have shown that a 3D-composite PENG can generate an output voltage near 65 V and an output current near 75 nA when 15% stretching stress is applied. This PENG is highly flexible and can generate electricity when pressed, stretched or bent [105]. Although PENGs have not been widely used for wearable sensors, this technology has ample potential for future breakthroughs and has been applied to further miniaturize conventional energy harvesters [106]. Additionally, this technology can potentially be integrated with other types of energy harvesting mechanisms [107].

Furthermore, piezoelectricity can be harvested from the human body irrespective of time or location [108]. The design of garments plays a vital role in energy collection, since clothing covers the human body as it moves [109], and an optimal design is needed to achieve mechanical deformation for efficient energy harvesting. The efficiency of piezoelectric energy harvesting depends on the properties and design of the textile being considered [110]. However, in addition to the garment texture, human factors such as the frequency of movement or the type of body part moving also play significant roles in the efficiency of piezoelectric energy harvesting [111].

PENGs have significant advantages over other power generation devices; for example, they are generally flexible and thus can be used for a broad range of applications. Table 8 lists some examples of PENGs that have been used to power wearable sensors. In real applications, the system supplying power should not be too large or complex in structure. In tradition, PENGs produce an AC voltage; as such, additional rectifiers are needed to convert the AC voltage to DC voltage. At the same time, human motion is typically of low frequency and not predictable; therefore, frequency up-conversion devices are also needed. These extra modules can lead to bulky and less-flexible generators. Recently, non-resonant piezoelectric energy harvesting techniques have been proposed to offset these defects. Bassani et al. proposed non-resonant macro-fiber composites in harvesting mechanical energy from human joint movements [112]. Benefiting from the netlike electrodes that can introduce a transverse mode inside fibers, P2 type macro-fiber composites used in this work are more suitable for energy harvesters compared to P1 types. These P2 type devices can also gain higher capacitance and generate more charge under the same strain conditions. As a result, both periodic and non-periodic movement can be made use of to generate electricity because the harvester’s power output depends on the bending velocity. On the other hand, walking with specific frequency is not necessary, and any motion that involves knee-bending can be used to gain energy. Another work demonstrated by Tuncel et al. built a theoretical model, and performed experiments to assess the amount of energy their harvester scavenged from human body joint motions [113]. The authors applied two kinds of macro-fiber composite patches and performed 16 groups of experiment to validate their theoretical model. The device can tolerate a large degree of bending (60° and 90° were tested) and can generate 13 μW average energy level for walking motions when attached to both knees. Cha and his group developed piezoelectric energy harvester to scavenge mechanical energy from finger’s clicking mouse [114]. The device is sealed onto gloves and tested by a robot finger under both one-click and double-click of a mouse. The maximum energy gained is in 1~10 nJ for 30–70 MΩ load resistance. In summary, it is promising to study further how to minimize the influence of unpredictable human motion and how to dispense the use of AC-DC converters. Further study is expected of the relationship between internal impedance, motion frequency, motion intensity and electric power.

#### 2.6.4. Triboelectric Nanogenerators

Different from electrostatic energy harvesters, TENGs generate surface static charge by contact electrification. When two materials with different electron affinities contact each other, there will be opposite electrostatic charges on the joint surface. Mechanical disturbance can build electric potential difference, which will then establish electric current through the contact surface. Based on this, TENGs can have broad application fields, given that we generate large amounts of wasted mechanical energy in daily activities. TENGs are highly flexible and stretchable. They can be attached to the elbow (armbands), legs (kneepads), wrists, fingers or feet (shoes) to harvest waste mechanical energy from human motion or the ambient environment. The combination of electrical textiles, TENGs and clothing can be promising in commercial applications. Table 9 provides some examples of TENG-powered biosensors.

TENGs have been used to power biosensor systems, wearable devices and electronic skin. Single fiber TENGs can provide an open circuit voltage of 140 V and a short circuit current density of 0.18 µA/cm. With an optimum load resistance of 320 MΩ, these TENGs can provide a power of 5.5 µW [121]. Non-texturized triboelectric devices can be worn on the skin to monitor electrophysiological signals, body temperature and hydration levels. Nanotexturized triboelectric devices can also measure electrophysiological signals and simultaneously convert imperceptible time-variant motions of the body into electrical signals. This process enables the self-powered monitoring of respiration, swallowing and arterial pulses [122]. Recently, fire-resistant and self-extinguishing TENGs with a stable electrical output under 200 °C have been proposed for special users such as firefighters [123]. These TENGs can be integrated into gloves or shoes and generate power by users walking, running or falling down.

AC voltage generated by TENGs needs to be converted into DC voltage. The extra circuit will increase the complexity of wearable and portable devices. To be applied to clinical medicine, they are expected to be sensitive enough to utilize tiny mechanical motions such as arterial pulsation.

## 3. Hybrid Energy Solutions

The combination of different energy sources allows for the generation of a larger amount of power and therefore increases the PCE of the energy system. A typical hybrid energy harvesting system consists of the energy harvester module, energy storage system and wearable sensor module. The remainder of this section introduces the currently most-popular hybrid systems.

### 3.1. Combination of Solar and Thermoelectric Energy Sources

Solar energy sources have a higher spectrum coverage for higher energy conversion. However, solar cells do not make full use of the photons outside of their band gap energy. Photons outside of the solar cell’s band gap energy are converted into waste heat.

**Figure 3:**
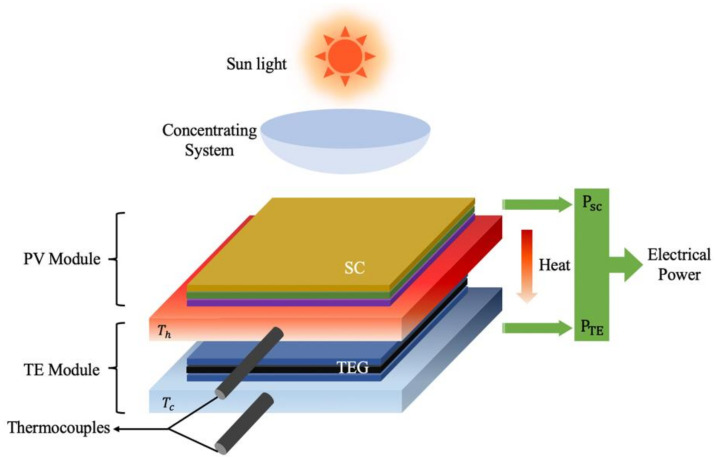
Schematic of a hybrid solar cell/TEG system. PV: photovoltaic; SC: solar cell; TE: thermoelectric; TEG: thermoelectric generator; P_sc_: electrical power generated by solar cell; P_TE_: electrical power generated by thermoelectric generator. T_h_: temperature of the TEG hot plate T_c_: temperature of the TEG cold plate (adapted from [131]).

Figure 3 shows a diagram of a conceptual photovoltaic and thermoelectric (PV-TE) system. As mentioned above, an increase in temperature will lead to a decrease in the PCE. Therefore, the combination of TE and PV energy systems could help broaden the use of these technologies and increase the total output power [127]. The two technologies can be combined effectively if there is a significant temperature difference across the thermoelectric module with contrasting flow of heat [128]. A PV-TE system that will be exposed to concentrated thermal radiation can be fabricated with optimized thermal management characteristics. Research collaborations have been conducted that allowed the theoretical and numerical calculation of heat flow and temperature distribution to determine the amount of energy generated from such a system [129]. Additionally, a copper plate introduced in the system as a thermal concentrator guaranteed that a difference in temperature on both sides of the thermoelectric module was obtained [130]. Due to the additional electrical energy generated from the TEG, the developed PV-TE hybrid system can achieve a theoretical efficiency of 23%, which is higher than that attainable by either single system. In wearable applications, device flexibility and indoor performance is also of great significance. One work demonstrated a flexible PV-TE bracelet to realize self-sustainability by harvesting solar energy and wasted human body heat. [46] This PV-TE cell can support a camera, a microphone, an accelerometer and temperature sensors under indoor illumination conditions. 

The optimal power output of a PV-TE hybrid device is nearly equal to the combined maximum power outputs of the individual PV and TE devices. One work suggested that the power supplied by an individual TEG or solar cell is not sufficient to light a commercial light-emitting diode (LED) with a 1.8 V turn-on voltage [132], while the PV-TE hybrid system can support this light. At the same time, lossless coupling between PV and TE devices is feasible, and the total PCE can be raised from 12.5% to 16.3% with only a 15 °C temperature gradient by simply incorporating a TE device with a PV system [133]. However, this hybrid generator is rigid and thus less compatible with wearable sensors than single PV or TE systems.

Additionally, TEG can produce an electrical energy amounting to 648 joules in 90 min even when there is no sunlight [134]. Since the system is cost effective and power efficient, it is desirable and economical as an energy source for wearable sensors.

### 3.2. Combination of TENGs and Solar Cells

**Figure 4:**
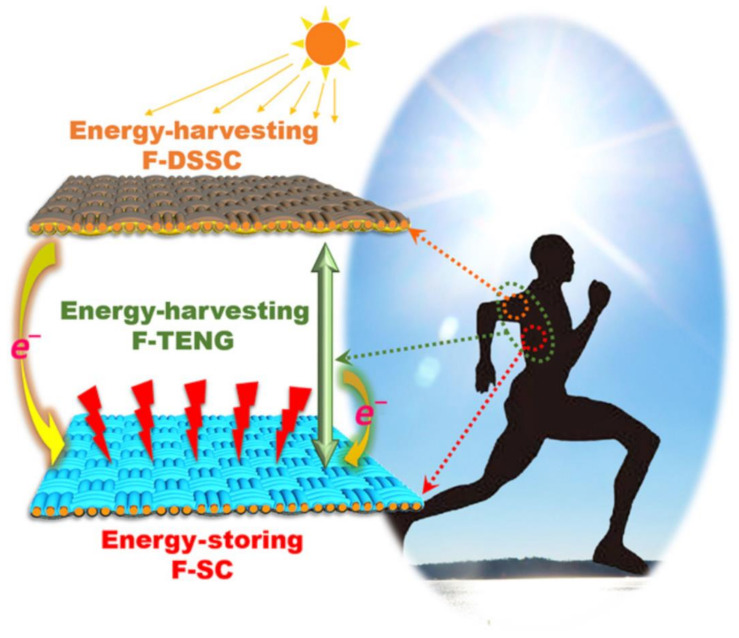
Schematic of the fiber-based, self-charging power system developed by Wen et al. [134], which is composed of an F-TENG, an F-DSSC energy harvesting fabric, and an F-SC energy-storing fabric. F-TENG: fiber-shaped triboelectric nanogenerator; F-DSSC: fiber-based, dye-sensitized solar cell; F-SC: fiber-based supercapacitor (adapted from [134] Courtesy of Dr. Zhong Lin Wang, with permission from the Publisher).

A hybrid self-charging textile capable of simultaneously collecting solar and body motion energy has been recently introduced, with the energy collected being stored in a supercapacitor [134]. Figure 4 shows a schematic of this self-charging textile, which consists of a double layer structure. It contains F-DSSC as the top layer to harvest sunlight energy and bottom F-SC layer to store energy. The connection of this F-DSSC and F-SC forms the F-TENG module that can make use of body motion energy. In this system, both solar and mechanical energy are converted into electricity. Solar radiation is converted into electrical energy by fiber-shaped dye-sensitized solar cells, and body motion is converted into electrical energy by fiber-shaped TENGs. This electrical energy is further converted to chemical energy in fiber-shaped supercapacitors.

Due to its all-fiber-shaped structure, the proposed self-charging textile can be easily integrated with electronic textiles to manufacture smart clothing to sustain the operations of wearable electronics and sensors.

### 3.3. Combination of Electromagnetic and Thermal Effects

A common strategy used by ESs to harvest energy is the utilization of electromagnetic effects. Scavengers can also be based on the Seebeck effect. Because the temperature can vary within metals and semiconductors, a voltage drop is observed across these materials. The most suitable method for measuring electromagnetic effects involves thermocouple technology. The two materials are integrated while maintaining their junctions at different temperatures. ESs that are based on this approach have many parallel thermocouples linked through an electrical connection to yield a thermopile [135]. Additional elements, such as radiators, may be used to transfer heat to the thermopile legs so as to substantially increase efficiency.

### 3.4. Combination of TENGs and PENGs

The TENG-PENG hybrid system can harvest mechanical energy from human motion or the ambient environment. One TENG-PENG system was able to successfully power a commercial digital watch and a temperature-humidity meter using wind as the energy source [136]. This type of hybrid generator can also be made portable and has been used to generate power for cell phones from hand vibrations [137].

In general, hybrid generators have fewer applications than single source generators because they are generally less flexible; however, they are able to offset the limitations caused by harvesting energy from a single source. To realize high compatibility with various applications, a good hybrid energy harvester should be of high flexibility. Table 10 lists some examples of flexible hybrid power generators that have been applied or can be used to power different kinds of wearable sensors.

## 4. Comparison of Various Energy Sources

Table 11 compares the various energy sources that can be used to power wearable sensors. These energy sources can act in single format or in combination; their operation and performance make them suitable for a variety of applications, and they can be used to power many different kinds of sensing devices. The suitability of a given energy source for a given application depends on the ambient environment, continuous sensing and sensing frequency requirements, the target analyte being detected, the cost, and human behavior, among many other factors. For any given application, chances are there exists a solution to power a dedicated wearable sensor to satisfy the performance requirements.

## 5. Summary and Conclusions

Sensing technologies have gained much popularity in health monitoring over the past few years. Wearable sensors dynamically and noninvasively measure biochemical markers found in biological fluids, including sweat, tears and interstitial fluids. Recently, the noninvasive monitoring of biomarkers such as metabolites, bacteria and hormones via electrochemical and optical biosensors has become a research hotspot. Researchers and scientists have miniaturized composite biosensors, transmission systems and microfluidic sampling platforms and integrated these modules into single wearable devices. However, these wearable sensing components cannot operate without energy sources.

Various technologies have been introduced to offer a wide range of energy solutions that allow these wearable microsystems to function as designed. In this review, we report the various energy solutions that have been used to provide either a constant or temporal power supply to wearable devices. We present the latest developments in solar cell, BFC, thermo-electric, triboelectric, piezoelectric, RF energy harvester and WPT technologies, as well as hybrid systems that combine these single energy sources. Each technology has advantages and disadvantages. In general, there are many single-format energy generators that perform well in powering wearable sensors. These generators are more mature and compatible with the human body and are less expensive than hybrid energy-harvesting systems. However, to enhance the PCE of the system, hybrid power generators are promising solutions.

Wearable sensors need to be flexible, twistable and durable, and must conform well to the human body. Most single-source energy generators and some hybrid generators meet these requirements; however, only a small proportion can function without the need for batteries as energy storage units. Therefore, additional research is needed to determine the capabilities of generators, especially hybrid generators, to supply stable and continuous power for wearable biosensors. Currently, the performance of these generators is simply measured by evaluating their electrical properties. Furthermore, a high PCE and low toxicity are of great importance. According to the research reviewed in this paper, optimizing the geometric structure and material fabrication process and developing new materials are the main approaches to improve the performance of energy generators. With continued technological advancements, further improvements in the available energy solutions for wearable sensors are achievable, and enhanced systems can offer permanent and reliable power supplies.

## Figures and Tables

**Table 1:** Parameters in this article.

Symbol	Meaning	Symbol	Meaning
$V_{oc}$	Open circuit voltage	$p_{o}$	Output power density
$V_{o}$	Output voltage	$R_{L}$	Load resistance
$J_{SC}$	Short circuit current	ΔT	Temperature difference
$P_{o}$	Output power	T	Temperature

**Table 2:** Comparison of different types of batteries.

Battery	Working Voltage [V]	Specific Energy		Specific Power	Life Cycle
		Gravimetric [W · h/kg]	Volumetric [W · h/L]		
Li-ion	3.7	~200	~550	High	>1000
Ni-MH	1.25~1.10	~100	<500	Low	500~1000
Ni-CD	1.25~1.00	<40	<100	High	300~2000
Ni zinc	1.60~1.40	~100	<400	High	<1000
Alkaline	1.3~1.0	100	<300	Medium	<30

Li-ion: Lithium-ion; Ni-MH: Nickel metal hydrogen; Ni-CD: Nickel chromium; Ni zinc: Nickel zinc.

**Table 3:** Typical low-modulus and flexible substrate wearable sensors powered by batteries.

Battery Chemistry	Voltage (V)	Capacity (Mah)	Sensor’s Target	Ref.
Li-ion polymer battery	3.7	150	Glucose, lactate, pH, temperature	[19]
Li-ion polymer battery	3.7	105	Glucose, lactate	[20]
Thin film Li-ion battery	N/A	N/A	Lactate	[21]
Li-ion battery	3.0	100	Lactate, potassium	[4]
Li-Po battery	N/A	N/A	OP nerve-agent compounds	[22]
Li coin battery	3.6	180	Lactate, Li ions	[23]
Button cell battery (Type: N/A)	1.5, 3.0	N/A	$H_{2}O_{2}$	[24]
Seat-type cell battery (Type: N/A)	N/A	25	Pressure	[25]

Ref: Reference; OP: organophosphate; Li: lithium; Po: polonium.

**Table 4:** Typical examples of flexible solar cells.

Device Structure	Fill Factor	$V_{oc}\;[V]$	$J_{SC}$ [mA · cm^−2^]	PCE	Ref.
FTO | co-dopped $TiO_{2}$ | perovskite | spiro-OMeTAD | Au	0.701	1.18	23.34	19.44%	[31]
MgF2 | Willow Glass | TCO | $SnO_{2}$ | FAMACs | Spiro-MeOTAD | $MoO_{x}$ | Al	0.752	1.084 V	22.16	18.1%	[32]
PET | IZO | PEDOT: PSS | perovskite | C_60_ | BCP | Ag	0.6011 ± 0.04	0.96 ± 0.04	18.05 ± 0.68	10.39 ± 0.41%	[33]
ITO | F-$TiO_{2}$ | KCsFAMA perovskite | spiro-OMeTAD | Au	0.7703 ± 0.019	1.17 ± 0.02	22.90 ± 0.61	20.66 ± 0.97%	[34]
ITO | PEDOT: PSS | perovskite | PCBM | Ag	0.745	0.75	27.8	15.59%	[35]
Glass | ITO | $SnO_{2}$ MaPbI_3_ | Carbon	0.61	0.95	22.94	13.08%	[36]
Glass | PEDOT: PSS (PH1000) | PEDOT: PSS (P VP AI4083) | PM6: Y6 | PFN-Br | Al	0.7729 ± 0.006	0.840 ± 0.010	23.92 ± 0.15	14.66 ± 0.24%	[37]

PCE: power conversion efficiency; Ref: Reference; FTO: Fluorine-doped tin oxide; $TiO_{2}$: titania; spiro-OMeTAD: 2,2′,7,7′-Tetrakis[N,N-di(4-methoxyphenyl)amino]-9,9′-spirobi- fluorene; TCO: transparent conductive oxide; Al: aluminum; PRT: poly(ethylene terephthalate); IZO: indium zinc oxide; PEDOT:PSS: poly(3,4- ethylenedioxythiophene) polystyrene sulfonate; BCP: bathocuproine; Ag: silver; ITO: indium tin oxide; F-$TiO_{2}$: $TiO_{2}$ nanocrystals; Gr: graphene; GO: graphene oxide; KCsFAMA: K_0.025_CS_0.05_FA_0.93_MA_0.12_Pbl_0.55_Br_0.45_; PCBM: [6,6]-Phenyl-C61-butyric acid methyl ester; PEN: polyethylene naphthalate; Ag-NW: silver nanowire; Ti: titanium; Pt: platinum; Cu: copper.

**Table 5:** Comparison of different flexible TEGs.

TE Material	Temperature	$P_{o}(\mathrm{or}p_{o})$	Ref.
n-type $Ag_{2}Se$	$T_{cold}=RT\;\Delta T=30\;K$	6.6 μW cm^−^	[64]
Ag-modified Bi_0.5_Sb_1.5_Te_3_	$T_{cold}=RT\;\Delta T=5,\;15,\;30,\;40,\;50,\;60\;K$	$12.4\;\mu W\cdot {\mathrm{cm}}^{-1}K^{-2}$ at 300 K	[65]
Bi_0.5_Sb_1.5_Te_3_ and Bi_2_Sb_0.3_Te_2.7_	ΔT=5~35 K	0.1~10 nW	[63]
Bi_2_Te_3_ grains	$T_{\mathrm{hot}}=307\;K,\;T_{\mathrm{air}}=293\;K$	153 μW · cm^−2^	[62]
(D-A)-type polymer/few-walled CNTs	ΔT= 20 K	210 nW	[66]

Ref: Reference; TE: thermoelectric; CNT: carbon nanotube; PI: polyimimide; PF: power factor; PDMS: polydimethylsiloxane; PVDF: polyvinylidene fluoride; D-A: Donner-accepter.

**Table 6:** Comparison of typical RF harvesters used for wearable devices.

Flexibility	Frequency	P_o_ (or p_o_)	PTE	Ref.
Flexible	2.45 GHz	NA	NA	[71]
Non-flexible	2.45 GHz	1~10 μW · cm^−2^	5.9~27.7%	[72]
Flexible	2.45 GHz	600 μW at 10 cm from source 80 μW at 60 cm from source	91%	[73]
Flexible	915 MHz & 1.85 GHz	NA	43.2% at −18 dBm input power	[74]
Flexible	5.2 GHz	NA	67% at +20 dBm input power	[75]
Flexible	0.868 GHz	NA	65.8% at +6 dBm input power	[76]

PTE: power transfer efficiency.

**Table 7:** Comparison of different BFCs that can be used to power wearable sensors.

Target	Sensitivity/LOD	P_o_ (or p_o_)	$V_{oc}$ or V_o_	Ref.
D-fructose	$3.82\pm 0.01\;mW\cdot {\mathrm{cm}}^{-2}\cdot {mM}^{-1}$	Pulse mode: 17.6 mW · cm^−2^ Constant load: 3.8 mW · cm^−2^	NA	[87]
Exosomes in cancer cells	300 particles $\cdot mM^{-1}$	619 μW · cm^−2^	(V_oc_) 0.46 V	[88]
Glucose	64.97 μA · cm^−2^ · mM^−1^	1011.21 μW · cm^−2^	NA	[89]
Glucose	NA	2.24 mW · cm^−2^	$\left(V_{o}\right)$ ~0.3 V	[90]
Glucose	0.1 mM	31.3 μW · cm^−2^	($V_{oc}$) ~0.65 V	[91]

LOD: limit od detection; Ref: Reference; RNA: ribonucleic acid.

**Table 8:** Comparison of different PENGs.

Form	Material	Periodical Pressure:	$R_{L}$	P_o_ (or p_o_)	Ref.
Flexible film	MASnBr_3_-PDMS	0.5 MPa/5 Hz	6 MΩ	~75.52 μW · cm^−2^	[115]
Fibers	PTrEE-PVDF	NA	NA	1.35 μW · cm^−3^	[116]
3D composite foam	(Sm-PMN-PT)-PDMS	NA	NA	11.5 μW · cm^−2^	[117]
Flexible film	MASnl_3_-PDMS	0.5 MPa/1 Hz	NA	21.6 μW · cm^−2^	[118]
Flexible composite	FAPnBr_3_ NPs/PVDF	0.5 MPa/5 Hz	200 kΩ	27.4 μW · cm^−2^	[119]
Flexible thin film	AIN buffer layer/$Al_{x}Ga_{1-x}N$ interlayer/top GaN layer	NA	5 MΩ	167 μW	[120]

Ref: Reference; MASnBr_3_: methylammonium tin bromide; SM-PMN-PT: samarium-doped Pb (Mg_1/3_Nb_2/3_) O_3_-PbTiO_3_; PDMS: polydimethylsiloxane; Pb: lead; PTrEE: polyrifluoroethylene; PVDF: polyvinylidene fluoride; MASnl_3_: methylammonium tin iodide; NPs: nanoparticles; AlN: aluminum nitride; GaN: gallium nitride.

**Table 9:** Comparison of various TENGs.

Form	Sensor Application	$R_{L}$	P_o_ (or p_o_)	Ref.
All-nanofiber-based TENG	Human movement monitoring	4610 MΩ	48.6 μW · m^−2^	[124]
Flexible chip	Detecting $NH_{3}$ ammonia at RT	46.2 MΩ	10.84 W · m^−2^	[125]
Electronic skin	Tactile sensing	140 MΩ	2.9 μW · cm^−2^	[126]

Ref: Reference; NH_3_ ammonia; RT: room temperature.

**Table 10:** Performance of latest flexible hybrid energy harvesting systems.

EH Type	EH Type	Device Type	Energy Storage System	P_o_ (or p_o_)	Comment	Ref.
PV-TE	Wristband	Medical sensor for Temperature Heartbeat Blood oxygen saturation Body acceleration	Super capacitor	PV: 207 mW TEG: 50 mW at ΔT = 20 K	Can support the integration of multiple medical systems.	[138]
PV-TE	Bracelet	NA	NA	NA	In helical structure. High stretchability and stability.	[139]
PV-TE	Wristband	NA	NA	23.1 μW at ΔT=70 K	ΔT=70K is very high in wearable applications	[140]
				4.48 μW for TEG at ΔT=30 K		
PV-TE	Bracelet	Data acquisition from the on-board camera and multiple sensors Visualization and wireless connectivity	Battery	550 μW for PV in door 250 μW for TEG at ΔT=5 K	Tested the indoor performance of PV section	[46]
PV-PENG	NA	NA	Super capacitor	0.97 W/cm^3^	Power conversion efficiency: 0.13%Stable output performance	[141]
TENG-PENG	Flexible substrate	LED lights	Light LED without storage section	151.42 · W/cm^2^	High Sensitivity against tiny body motion	[142]
TENG-PENG	Flexible slice	NA	NA	V_oc_: 5.2 V J_sc_: 500 nA	High Sensitivity against tiny motion	[143]

EH: energy harvester; Ref: Reference; TENG: triboelectric nanogenerator; PV: photovoltaic; TE: thermoelectric; PENG: piezoelectric nanogenerator; V_oc_: open circuit voltage; J_sc_: short circuit current.

**Table 11:** Characteristics of various energy sources for wearable sensors.

Power Source	Advantages	Drawbacks	Comments
Batteries	Provide the greatest amount of power	Need replacement or frequent charge. not flexible for wearable applications. toxic	Suitable for applications that require larger components and more power
Perovskite solar cells	Flexible; lightweight; compatible with wearable applications [39]	Require light	Exhibit higher efficiency than that of organic flexible solar cells [34]
Biofuel cells	Sensing and power modules are integrated for enhanced miniaturization; no need for light, RF waves, or body motion [77]	Power density can be affected by the analyte concentration	Suitable for wearable sensors that monitor sweat [80]
Radio frequency harvesters	Reliable source of energy	Low power; need sufficient RF signal levels	Offer a more continuous and controllable source of power than chemical sources or solar cells [71]
Thermoelectric generators	No need for light or body motion; cost effective.	Low-power source [64]	Suitable for applications requiring continuous or uninterrupted monitoring
Triboelectric generators	No need for light or RF waves	Low-power source; require body motion [124]	Usually combined with other techniques to provide useful power [134]
Piezoelectric generators	No need for light or RF waves	Low-power source; require body motion [102]	Usually combined with other techniques to provide useful power [134]
Hybrid techniques	Higher levels of power production	More complex in terms of system design and materials; power management modules are generally needed [137]	More cost and power effective than single source technologies [132]
